# Supplementary material for: Preferences and patterns of response to public health advice during the COVID-19 pandemic
Source: Sci Rep. 2021 Nov 4;11:21700. doi: 10.1038/s41598-021-01186-6 (PMC8569172; doi:10.1038/s41598-021-01186-6)
Supplement: Supplementary file 1 — Supplementary Information. [file 41598_2021_1186_MOESM1_ESM.pdf]

**Preferences and patterns of response to public health advice during the COVID-19 pandemic**

## Supporting Information

We recruited a total of 1,061 participants on Amazon Mechanical Turk (MTurk). Participation was limited to people with a US MTurk account, who had completed more than 100 prior HITs with an approval rate greater than 95%. Participants could take part in this study - across all its variants - only once. Of these participants, 409 self-identified as female, 623 as male, 3 as non-binary, and the remainder did not respond. Their self-reported ages ranged between 18 and 78, with a mean age of 36.1 (median = 34). Participants were paid a flat rate of either $2.50 for completing the study variants in which information about expected infection rates were provided (median time spent on these variants of the study was 3 minutes 24 seconds), or $2 for completing study variants in which information about expected infection rates were not provided (median time spent on these variants of the study was 5 minutes 14 seconds). This difference was due to the extended time required to read each of the explanatory paragraphs and infection rate examples.

Participants were given an attention check question, in which we asked them to identify the proportion of people that did not adhere to any advice who would be infected. This information was repeated in each of the explanatory text paragraphs, and so could be easily answered without reference to outside knowledge. Such attention checks are commonly used to address potential concerns over data validity in studies where participants are recruited through MTurk [Hauser 2016, Landers 2015]. We did not include the attention check question in study variants that did not include infection rate information. For these participants we relied solely on the more basic attention check of filtering for minimum time spent on the study (one minute) to exclude those participants who had simply rushed through the study without thought or attention. This inclusion criterion was also applied to participants who had passed the attention check question.

Study 1

In Study 1a participants' preferences between strict and relaxed advice were compared, without information about the likelihood of infection resulting from either of these choices (N=138).

In Study 1b we sought to separate participants' preferences for advice from their unknown assumptions about the advice's expected effectiveness. Here, we examined what effect differences in infection expectancy across the advice strategies had on participants' choices. Participants were presented the same scenario as in Study 1a, but the projected infection rates for the 'relaxed advice' were manipulated such that infection rates among those who received the strict advice are *lower/higher/equal* than the infection rate among those who receive the relaxed advice. In the "lower" condition (N=68), the projected infection rates for 'strict advice' (1,420 of the 10,000 city residents) was lower than for 'relaxed advice' (1,630); in the "higher" condition (N=71)) the projected infection rates for 'strict advice' (1,525) was higher than for 'relaxed advice' (1,420); and in the "equal" condition (N=128) infection rates were the same

(1,525 out of 10,000 residents) whether advice was strict or relaxed. The order in which the ‘strict advice’ and the ‘relaxed advice’ options appeared on the screen was also randomized, so that for roughly half of participants the ‘strict advice’ option was presented first and the ‘relaxed advice’ option second, and for the remaining half this was reversed.

Study 2

With three options for participants to choose from, we varied the projected infection rates randomly so that for roughly one third of participants the total projected infection rates for presenting relaxed advice (1,420 out of the 10,000 city residents in the example text) and the informed choice advice (1,435) were lower than that for strict advice (1,525) (N=90), for another third of participants they were exactly equal (1,525) (N=89), and for the final third of participants the projected infection rates for relaxed advice (1,630) and informed choice advice (1,605) were higher than that for strict advice (1,525) (N=90). We again randomized the order in which the strict and relaxed advice options appeared on the screen.

Text presented to participants in each study variant, reflecting the different conditions - adherence rates expected infection rates variations:

STRICT ADVICE
Advice that is medically best for preventing infection.
“Wear a mask at all times outside the home. Keep 6 feet away from others.”

- Among people who comply with this advice there is an average infection rate of 1% within 1 month, and on average 25% of the public are likely to comply with this advice. The infection rate among the remainder who do not comply with this advice is 20% within 1 month.
  Example: In a city with a population of 10,000 people, it is likely that 2,500 will comply with this advice and on average 25 of these will be infected. The remaining 7,500 people are likely not to comply with this advice, and on average 1,500 of these will be infected. In this scenario, it is likely that a total of 1,525 people out of the city’s population of 10,000 will be infected within 1 month.

Relaxed advice -
Useful advice that is more likely to be adopted by a larger proportion of people
"Wear a mask in enclosed public spaces where it is not possible to maintain a safe distance from others."

Three expected infection variations:

- Among people who comply with this advice there is an average infection rate of 10% within 1 month, and on average 47.5% of the public are likely to comply with this advice. The infection rate among the remainder who do not comply with this advice is 20% within 1 month.
  Example: In a city with a population of 10,000 people, it is likely that 4,750 will comply with this advice and on average 475 of these will be infected. In contrast, the remaining 5,250 are likely not to comply with this advice, and on average 1,050 of these will be infected. In this scenario, a total of 1,525 people out of the city’s population of 10,000 will be infected within 1 month.

- Among people who comply with this advice there is an average infection rate of 10% within 1 month, and on average 37% of the public are likely to comply with this advice. The infection rate among the remainder who do not comply with this advice is 20% within 1 month.
  Example: In a city with a population of 10,000 people, it is likely that 3,700 will comply with this advice, and on average 370 of these will be infected. The remaining 6,300 are likely not to comply with this advice, and on average 1,260 of these will be infected. In this scenario, a total of 1,630 people out of the city's population of 10,000 will be infected within 1 month.

- Among people who comply with this advice there is an average infection rate of 10% within 1 month, and on average 58% of the public are likely to comply with this advice. The infection rate among the remainder who do not comply with this advice is 20% within 1 month.
  Example: In a city with a population of 10,000 people, it is likely that 5,800 will comply with this advice, and on average 580 of these will be infected. The remaining 4,200 are likely not to comply with this advice, and on average 840 of these will be infected. In this scenario, a total of 1,420 people out of the city's population of 10,000 will be infected within 1 month.

Informed choice - text presented to participants:
Offer the public both types of advice.
"The best medical advice is to wear a mask at all times outside the home and keep 6 feet away from others. However, wearing a mask in enclosed public spaces where it is not possible to maintain a safe distance from others, also offers some protection from infection."

Three expected infection variations:

- If both sets of advice are offered we would expect reduced adherence with each in comparison to when offered alone. Among people who comply with the best medical advice there is an average infection rate of 1% within 1 month, and on average 15% of the public are likely to comply with this advice. Among people who comply with the advice more likely to be adopted there is an average infection rate of 10% within 1 month, and on average 37% of the public are likely to comply with this advice. The infection rate among the remainder who do not comply with either advice is 20% within 1 month.
  Example: In a city with a population of 10,000 people, it is likely that 1,500 will comply with the medically-best advice, and on average 15 of these will be infected. A further 1,900 are likely to comply with the advice more likely to be adopted, and on average 190 of these will be infected. The remaining 6,600 are likely not to comply with either advice, and on average 1,320 of these will be infected. In this scenario, a total of 1,525 will be infected within 1 month.

- If both sets of advice are shown we would expect reduced adherence with each in comparison to when offered alone. Among people who comply with the best medical advice there is an average infection rate of 1% within 1 month, and on average 15% of the public are likely to comply with this advice. Among people who comply with the advice more likely to be adopted there is an average infection rate of 10% within 1 month, and on average 11% of the public are likely to comply with this advice. The infection rate among the remainder who do not comply with either advice is 20% within 1 month.
  Example: In a city with a population of 10,000 people, it is likely that 1,500 will comply with the medically-best advice, and on average 15 of these will be infected. A further 1,100 are likely to comply with the advice more likely to be adopted, and on average 110 of these will be infected.

The remaining 7,400 are likely not to comply with either advice, and on average 1,480 of these will be infected. In this scenario, a total of 1,605 will be infected within 1 month.

- If both sets of advice are shown we would expect reduced adherence with each in comparison to when offered alone. Among people who comply with the best medical advice there is an average infection rate of 1% within 1 month, and on average 15% of the public are likely to comply with this advice. Among people who comply with the advice more likely to be adopted there is an average infection rate of 10% within 1 month, and on average 28% of the public are likely to comply with this advice. The infection rate among the remainder who do not comply with either advice is 20% within 1 month.
  Example: In a city with a population of 10,000 people, it is likely that 1,500 will comply with the medically-best advice, and on average 15 of these will be infected. A further 2,800 are likely to comply with the advice more likely to be adopted, and on average 280 of these will be infected. The remaining 5,700 are likely not to comply with either advice, and on average 1,140 of these will be infected. In this scenario, a total of 1,435 will be infected within 1 month.
